# Non-Hispanic White Mothers’ Willingness to Share Personal Health Data With Researchers: Survey Results From an Opt-in Panel

**DOI:** 10.2196/14062

**Published:** 2020-05-15

**Authors:** Adam Bouras, Eduardo J Simoes, Suzanne Boren, Lanis Hicks, Iris Zachary, Christoph Buck, Satvinder Dhingra, Richard Ellis

**Affiliations:** 1 Department of Health Management and Informatics University of Missouri-Columbia Columbia, MO United States; 2 Missouri University Institute for Data Science and Informatics University of Missouri-Columbia Columbia, MO United States; 3 Centre for Future Enterprise QUT Business School Queensland University of Technology Brisbane Australia; 4 Public Good Venture Limited Atlanta, GA United States

**Keywords:** surveys and questionnaires, confidentiality, electronic health records, fitness trackers, mobile applications, logistic models

## Abstract

**Background:**

Advances in information communication technology provide researchers with the opportunity to access and collect continuous and granular data from enrolled participants. However, recruiting study participants who are willing to disclose their health data has been challenging for researchers. These challenges can be related to socioeconomic status, the source of data, and privacy concerns about sharing health information, which affect data-sharing behaviors.

**Objective:**

This study aimed to assess healthy non-Hispanic white mothers’ attitudes in five areas: motivation to share data, concern with data use, desire to keep health information anonymous, use of patient portal and willingness to share anonymous data with researchers.

**Methods:**

This cross-sectional study was conducted on 622 healthy non-Hispanic white mothers raising healthy children. From a Web-based survey with 51 questions, we selected 15 questions for further analysis. These questions focused on attitudes and beliefs toward data sharing, internet use, interest in future research, and sociodemographic and health questions about mothers and their children. Data analysis was performed using multivariate logistic regressions to investigate the factors that influence mothers’ willingness to share their personal health data, their utilization of a patient portal, and their interests in keeping their health information anonymous.

**Results:**

The results of the study showed that the majority of mothers surveyed wanted to keep their data anonymous (440/622, 70.7%) and use patient portals (394/622, 63.3%) and were willing to share their data from Web-based surveys (509/622, 81.8%) and from mobile phones (423/622, 68.0%). However, 36.0% (224/622) and 40.5% (252/622) of mothers were less willing to share their medical record data and their locations with researchers, respectively. We found that the utilization of patient portals, their attitude toward keeping data anonymous, and their willingness to share different data sources were dependent on the mothers’ health care provider status, their motivation, and their privacy concerns. Mothers’ concerns about the misuse of personal health information had a negative impact on their willingness to share sensitive data (ie, electronic medical record: adjusted odds ratio [aOR] 0.43, 95% CI 0.25-0.73; GPS: aOR 0.4, 95% CI 0.27-0.60). In contrast, mothers’ motivation to share their data had a positive impact on disclosing their data via Web-based surveys (aOR 5.94, 95% CI 3.15-11.2), apps and devices designed for health (aOR 5.3, 95% CI 2.32-12.1), and a patient portal (aOR 4.3, 95% CI 2.06-8.99).

**Conclusions:**

The findings of this study suggest that mothers’ privacy concerns affect their decisions to share sensitive data. However, mothers’ access to the internet and the utilization of patient portals did not have a significant effect on their willingness to disclose their medical record data. Finally, researchers can use our findings to better address their study subjects concerns and gain their subjects trust to disclose data.

## Introduction

### Background

Advances in information communication, electronic health (eHealth), and mobile health technologies are increasingly used to access and collect personal health data. These have contributed to the expansion of research in health care and public health. The eHealth apps can be Web-based or mobile apps that include a range of features, such as tracking changes in health behaviors, chronic care management by health professionals or the patients themselves, or location tracking. In this regard, many eHealth studies have focused on adult women, and specifically mothers, who have often been used as proxy respondents for studying the diverse health care issues of their families [1]. Moreover, there has been an increasing interest among commercial organizations and researchers in recruiting mothers to gain insight into their behaviors while using the internet [2,3]. In 2014, 85% of white women in the United States used the internet [4]. In an eMarketer report [5], mothers, if that is true, more often requested immediate and continuous access to the internet, compared with total female consumers in general (53.9% vs 44.3%, respectively). Women, particularly mothers, tend to use the internet to look for health and medical information, get support from different sources, and search for other information needs [6,7]. Although research in the area of data sharing is increasing, many questions remain to be answered that mostly pertain to privacy and data-sharing preferences.

### Privacy and Data Disclosure

Privacy and data disclosure are considered serious challenges for health care researchers, and patients are hesitant about sharing their health data as they may expect a loss of privacy while sharing their personal information. This issue encourages patients to retain control over their personal information and disclosure of their data. In fact, patients undergo a cost-benefit analysis to assess different factors that influence their preferences and decisions to share their data, which is known as the privacy calculus [8-10].

Although a substantial body of literature has examined individuals’ willingness to share their health data, most of these studies have focused on health information exchanges in the general population [11-19]. These findings have created interest in how to solicit information from patients and maximize their participation in research studies. Most studies have found that data security and privacy preferences shape consumers’ attitudes toward health information exchanges [11,12,14-16,18-21]. As reported, consumers perceive health information exchanges to confer benefits, such as better coordination of care [18] and improvement of health care quality [14]. Accordingly, researchers have considered privacy control in the context of research to enhance the engagement of individuals and establish trust with the study participants.

### Objective

An attempt to find a solution to greater privacy control has increased the number of studies addressing privacy on the basis of differentiation between sensitive and less sensitive personal health data. In this regard, consumers have more choices to share their personal data with whom they feel more comfortable [22]. Therefore, innovative systems are being developed to give individuals more power to determine the researchers who are allowed to access their data and the type of data they are willing to share [23]. However, the granularity of data necessary for this level of privacy increases the burden on stakeholders (ie, researchers) and a priori market research, as well as market segmentation, can facilitate the assessment of consumers’ willingness to share their data. Accordingly, a number of studies have sought to gauge how sensitive consumers are about sharing their data and the amount of data they are willing to disclose. As a result, this study aimed to assess healthy white mothers’ attitudes toward the anonymity of their health information and their utilization of the Web-based patient portals and willingness to share different data sources with researchers.

## Methods

### Study Design

A total of 622 women were randomly selected from a commercial opt-in panel with several million US members. The participants were non-Hispanic white women with children from the Centers for Disease Control and Prevention–funded study on the care burden of mothers of children with autism spectrum disorder (ASD), implemented collaboratively by the Department of Health Management and Informatics of the University of Missouri School of Medicine and the Kennedy Krieger Institute’s Interactive Autism Network (IAN). The study compared care burdens and associated factors between a US representative sample of mothers and the sample of mothers of children with ASD from the IAN registry. As the proportion of nonwhite mothers in the IAN registry was very small, the design required the selection of a comparative US sample of white women raising children without disabilities. According to the sampling method of the original study, the samples in this study were representative of white mothers aged 25 years or older living in an urban area. The majority of the investigated individuals had an educational level of college (4 years) and were employed or self-employed.

### Survey Questionnaire

The research tool was a survey questionnaire with different domains related to attitudes and beliefs, trust in data sharing, data sharing through mobile phone apps and devices, internet use, and interest in future research, as well as questions related to caregivers and their children. The survey questionnaire consisted of 51 questions, of which 15 were selected for further analysis. The questions selected were related to attitudes and beliefs about data sharing, internet use, and interest in future research, sociodemographic information, and health questions about caregivers and their children.

### Outcome Variables

On the basis a review of empirical studies [7,24,25], mothers were assessed based on their preference for keeping their health data anonymous. As reported in other studies, patients are more willing to disclose their data when the data are anonymously shared with researchers [26]. In this regard, respondents were asked to answer the following question: “How important is it to you that your personal health information is kept anonymous?” To answer the questions, the respondents could choose from the following four choices: *extremely important*, *very important*, *somewhat important*, and *not at all important*. The *extremely important* and *very important* answers were grouped into an *extremely important* category for analysis.

Similarly, mothers’ familiarity with patient portals was investigated in this study, as familiarity with the source of health care data may influence patients’ willingness to share their data. For instance, a study conducted by the United States Department of Veterans Affairs indicated that veterans had a higher level of willingness to share their health information when they gained a higher level of familiarity with the utilization of a Web-based portal [24]. Therefore, it could be suggested that when the majority of the mothers are familiar with the patient portal, they will be more inclined to share their medical records with researchers [24]. Accordingly, respondents were asked about their familiarity with the patient portal and frequency of its use. The answer provided by the investigated participants could be one of the following alternatives: *never heard of it*; *heard of it but never used it*; *yes, once a month*; and *yes, more than once a month*. The answers were then dichotomized into two categories⸺*never used it* (combining never heard of it and heard of it but never used it) and *have used it more than once* (combining *yes, once a month* and *yes, more than once a month*).

The respondents were also required to answer the question, “Which type of health-related data are you willing to share anonymously with researchers?” They could choose from the following choices: *data from medical records*, *data you provide directly by completing an online survey*, *data you entered into a health-related mobile app or device*, *data collected automatically by mobile app or device*, and *GPS location data from a mobile device*. Answers to the following choices *data you enter into a health-related mobile app or device*, *data that is collected automatically by mobile app or device* and *data entered into or collected through mobile app or device* were considered as a positive category for sharing data for analysis.

### Independent Variables

To address respondents’ privacy calculus [26], two questions were included to measure respondents’ concerns and motivations for sharing their health data. The respondents were asked, “How concerned are you that your health record might be…”, to which they could select one of the following choices: *used to deny me health care benefits*, *used to limit my job opportunities*, *used without my knowledge*, and *stolen by unknown individuals or companies*. They could rate these choices as *very concerned*, *somewhat concerned*, *only a little concerned*, *not at all concerned*. If the answers provided to all the choices were *very concerned*, the score would be 4, and the response *not at all concerned* for all choices resulted in a total score of 16. Respondents with the total score <8 were categorized as *very concerned*, those with scores ranging from 8 to 11 as *somewhat concerned*, and individuals with scores >11 as *less concerned*.

Similarly, to assess mothers’ motivation to share data with researchers, the participants were asked, “What is your motivation for sharing your health information?” The mothers could select any of the following choices: *benefit future patients*, *contribute to science and research*, *trust in researcher organization, and a desire to contribute to the research they are doing*, *establish a good relationship with researcher organization*, and *other*. The obtained motivation score was the sum of the selected answer choices ranging from 0 to 5. A score of 0 meant that the respondent did not select any choices, and we categorized this respondent as *not motivated*. On the other hand, the score of 5 was indicative of mothers who selected all the answer choices, so they were *highly motivated*. In the next step, the motivation scores were divided into three levels. The individuals with motivation scores ≥1 were grouped into *less motivated*, whereas those with motivation scores between 1 and 3 were grouped into *somewhat motivated*, and motivation scores >3 were *very motivated*.

We dichotomized the variables of age (18-49 and >50 years), education (less than 4 years of college and 4 or more years of college), occupational status (self-employed and other occupational status), income level (household income ≤US $74,999 and ≥US $75,000), marital status (married and other status), age of the child/young adult (≤14 years and ≥15 years), number of children (1 child and more than 1 child), health status of the youngest child/adult, and mothers’ health status in general (excellent to good and fair to poor) based on their frequency distribution.

### Data Analysis

Data analysis was conducted using SAS (version 9.3). Moreover, the frequency analysis was carried out to describe the demographic characteristics of the surveyed mothers, their data-sharing preferences, and their privacy concerns [27]. Multivariate logistic regression was also conducted to investigate the associations of selected outcomes and a set of independent variables. In the models, the dependent variables were *desire to keep personal health information anonymous*, *use of patient portal*, and *willingness to share anonymous data with researchers regarding medical records, Web-based survey, health apps or devices, and GPS locator*. The independent variables in the models were age, educational status, occupational status, marital status, household income, mothers’ health status, child health status, health care provider (HCP) status, children’s age, number of children, use of internet on the mobile phone to access health information, mothers’ motivation to share their data, and mothers’ concerns about sharing health data. We generated odds ratios (ORs) with 95% Cis across levels of independent variables. *P* values <.05 were considered statistically significant at the 95% confidence level for the OR.

## Results

### Demographic Characteristics

Table 1 displays the demographic characteristics of non-Hispanic white mothers who responded to the survey questions. The survey results showed that the majority of the mothers were married (485/622, 78.0%) women aged between 18 and 49 years (444/622, 71.4%) who had an excellent-to-good health condition (560/622, 90.0%). Regarding the occupational status of the participants, a large number of the investigated mothers were employed or self-employed (452/622, 72.7%). More than half of the mothers had at least a 4-year college degree (347/622, 55.8%), with a yearly household income of over $75,000 (388/622, 62.4%). More than half of the mothers reported having 1 child (326/622, 52.4%), and the majority of their children or young adults were in excellent-to-good health status (594/622, 95.5%).

Mothers’ motivation to share their data was split nearly equally between less motivated (302/622, 48.6%) and motivated (320/622, 51.4%). The mothers were motivated to share their data to contribute to science (326/622, 52.4%) and to benefit patient health (387/622, 62.2%)—these results are available upon request.

The majority of the respondents were concerned about the misuse of personal health information (507/622, 81.5%). In fact, the respondents were very concerned that their data would be stolen by unknown individuals or companies (360/622, 57.9%) or if their health data would be used without their consent and knowledge (340/622, 54.7%)—these results are available upon request.

Respondents were more willing to share their data with the researchers provided through Web-based surveys (509/622, 81.8%) and collected through their mobile phones (423/622, 68.0%) compared with their medical record data (224/622, 36.0%) and GPS locations (252/622, 40.5%).

**Table 1:** Frequency of mothers’ demographic characteristics (N=622).

Demographics		Values, n (%)
**Age group (years)**		
	18-49	444 (71.4)
	>50	178 (28.6)
**Education**		
	Less than 4 years of a college degree	275 (44.2)
	4 or more years of college	347 (55.8)
**Occupational status**		
	Employed or self-employed	452 (72.7)
	Other occupational status	170 (27.3)
**Marital status**		
	Married	485 (78.0)
	Other marital status	137 (22.0)
**Household income (US$)**		
	≤74,999	234 (37.6)
	≥75,000	388 (62.4)
**Mothers’ health status**		
	Fair to poor	62 (10.0)
	Excellent to good	560 (90.0)
**Child health status**		
	Fair to poor	28 (4.5)
	Excellent to good	594 (95.5)
**HCP^a^ status**		
	I don’t have	52 (8.4)
	Have more than 1 HCP	190 (30.5)
	Yes, just 1 HCP	380 (61.1)
**Children’s age (years)**		
	≤14	381 (61.1)
	≥15	241 (38.7)
**Number of children**		
	One child	326 (52.4)
	More than one child	296 (47.6)
**Use of mobile phones**		
	Yes	553 (96.0)
	No	21 (3.4)
**Use of the internet to access health information^b^**		
	Yes	361 (58.0)
	No	251 (40.4)
**Mothers’ motivation for sharing their data**		
	Less motivated	302 (48.6)
	Somewhat motivated	248 (39.9)
	Very motivated	72 (11.6)
**Mothers’ concerns about the misuse of personal health information**		
	Less concerned	115 (18.5)
	Somewhat concerned	227 (36.5)
	Very concerned	280 (45.0)
**Utilization of patient portals**		
	Never used it	228 (36.7)
	Used it more than once	394 (63.3)
**Desire to keep personal health information anonymous**		
	Not at all	13 (2.1)
	Somewhat	169 (27.2)
	Extremely	440 (70.7)
**Willingness to share anonymous data from medical records**		
	Yes	224 (36.0)
	No	398 (64.0)
**Willingness to share anonymous data provided through a Web-based survey**		
	Yes	509 (81.8)
	No	113 (18.1)
**Willingness to share anonymous data entered into or collected by health-related app or device**		
	Yes	423 (68.0)
	No	199 (32.0)
**Willingness to share GPS location anonymously from a mobile app**		
	Yes	252 (40.5)
	No	370 (59.5)

^a^HCP: health care provider.

^b^48 participants did not respond to this question.

### Associations of Mothers’ Desire to Keep Their Health Information Anonymous and Independent Variables

The chi-square test results showed that the HCP status (*P*=.02), the use of the internet to access health information (*P*=.03), mothers’ motivation (*P*=.01), and concerns (*P*<.001) about sharing health data were all associated with mothers’ desires to keep their data anonymous (Multimedia Appendix 1). After adjusting for the demographic characteristics of mothers using multivariate logistic regression, only mothers’ concerns about sharing data were associated with the desire to keep health information anonymous (Table 2). Mothers who were very concerned and somewhat concerned were more than two times and four times more likely to keep their health information anonymous than less-concerned mothers (adjusted odds ratio [aOR] 4.77, 95% CI 2.85-7.96; aOR 2.50, 95% CI 1.52-4.1, respectively).

**Table 2:** Results of multivariate logistic regression regarding the association between mothers’ desire to keep their health information anonymous and a set of predictors, including their demographic characteristics.

Effect		aOR^a^ (95% CI)	*P* value^b^	
**Age (years)**				**.86**
	18-49	0.95 (0.55-1.64)		
	>50	1.00 (Reference)^c^		
**Educational level**				**.17**
	4 or more years of college	0.75 (0.5-1.13)		
	Less than 4 years of a college degree	1.00 (Reference)^c^		
**Occupational status**				**.20**
	Employed	1.34 (0.86-2.1)		
	Unemployed	1.00 (Reference)^c^		
**Marital status**				**.35**
	Married	1.27 (0.77-2.1)		
	Unmarried	1.00 (Reference)^c^		
**Income level (US $)**				**.42**
	≥75,000	1.19 (0.77-1.84)		
	≤74,999	1.00 (Reference)^c^		
**Health status**				**.93**
	Excellent to good	1.03 (0.52-2.03)		
	Fair to poor	1.00 (Reference)^c^		
**Child health status**				**.36**
	Excellent to good	0.61 (0.21-1.75)		
	Fair to poor	1.00 (Reference)^c^		
**HCP^d^ status**				**.50**
	More than 1 HCP	0.82 (0.37-1.84)		
	Just 1 HCP	0.69 (0.32-1.47)		
	No HCP	1.00 (Reference)^c^		
**Children’s age (years)**				**.99**
	≤14	1.00 (0.59-1.7)		
	≥15	1.00 (Reference)^c^		
**Number of children**				**.98**
	More than one child	1.01 (0.66-1.54)		
	One child	1.00 (Reference)^c^		
**Use of mobile phones**				**.14**
	Yes	2.06 (0.8-5.33)		
	No	1.00 (Reference)^c^		
**Use of the internet to access health information**				**.33**
	Yes	0.35 (0.04-2.91)		
	No	1.00 (Reference)^c^		
**Mothers’ motivation to share their data**				**.11**
	Somewhat motivated	0.66 (0.43-1)		
	Very motivated	0.66 (0.36-1.22)		
	Less motivated	1.00 (Reference)^c^		
**Mothers’ concern about sharing health data**				**<.001**
	Somewhat concerned	2.50 (1.52-4.1)		
	Very concerned	4.77 (2.85-7.96)		
	Less concerned	1.00 (Reference)^c^		

^a^Adjusted odds ratios (aORs) of reporting desire to keep health information anonymous from a multivariable logistic regression model, conditional on mothers’ motivation and concerns and all other characteristics

^b^*P* values from Type 3 analysis based on the Wald test.

^c^Reference group does not have CI.

^d^HCP: health care provider.

### Associations of Mothers’ Use of Patient Portals With Independent Variables

Bivariate analyses indicated that child health status (*P*=.03), status of HCP (*P*<.001), use of mobile phones (*P*<.001), use of the internet to access health information (*P*<.001), and mothers’ motivation to share data (*P*<.001) were statistically associated with mothers’ use of the patient portal (Multimedia Appendix 1). However, after adjusting for mothers’ demographic characteristics using multivariate logistic regression models, only HCP status and mothers’ motivation to share data had statistically significant associations with the utilization of the patient portal (Table 3). The likelihood of using patient portals increased four times for mothers who had 1 HCP (aOR 3.47, 95% CI 1.73-6.94) and more than one HCP (aOR 4.3, 95% CI 2.06-8.99). Similarly, very motivated mothers who were interested in sharing their health data used the patient portal twice as much as other investigated mothers (aOR 2.09, 95% CI 1.12-3.91).

**Table 3:** Results of multivariate logistic regression regarding the relationship between mothers’ use of the patient portal and a set of predictors, including mothers’ demographic characteristics.

Effect		aOR^a^ (95% CI)	*P* value^b^	
**Age (years)**				**.54**
	18-49	0.86 (0.52-1.41)		
	>50	1.00 (Reference)^c^		
**Education level**				**.92**
	4 or more years of college	0.98 (0.67-1.44)		
	Less than 4 years of a college degree	1.00 (Reference)^c^		
**Occupational status**				**.54**
	Employed	0.87 (0.57-1.35)		
	Unemployed	1.00 (Reference)^c^		
**Marital status**				**.91**
	Married	0.97 (0.6-1.58)		
	Unmarried	1.00 (Reference)^c^		
**Income level (US $)**				**.11**
	≥75,000	1.40 (0.93-2.12)		
	≤74,999	1.00 (Reference)^c^		
**Health status**				**.70**
	Excellent to good	0.88 (0.46-1.68)		
	Fair to poor	1.00 (Reference)^c^		
**Child health status**				**.13**
	Excellent to good	0.43 (0.14-1.3)		
	Fair to poor	1.00 (Reference)^c^		
**HCP^d^ status**				**<.001**
	More than one HCP	4.3 (2.06-8.99)		
	Just one HCP	3.47 (1.73-6.94)		
	No HCP	1.00 (Reference)^c^		
**Children’s age (years)**				**.32**
	≤14	1.29 (0.79-2.11)		
	≥15	1.00 (Reference)^c^		
**Number of children**				**.52**
	More than one child	1.14 (0.76-1.72)		
	One child	1.00 (Reference)^c^		
**Use of mobile phones**				**.15**
	Yes	2.01 (0.77-5.2)		
	No	1.00 (Reference)^c^		
**Use of the internet to access health information**				**.26**
	Yes	2.27 (0.55-9.32)		
	No	1.00 (Reference)^c^		
**Mothers’ motivation to share their data**				**.006**
	Somewhat motivated	1.75 (1.18-2.59)		
	Very motivated	2.09 (1.12-3.91)		
	Less motivated	1.00 (Reference)^c^		
**Mothers’ concern about sharing health data**				**.26**
	Somewhat concerned	1.43 (0.85-2.39)		
	Very concerned	1.46 (0.88-2.42)		
	Less concerned	1.00 (Reference)^c^		

^a^Adjusted odds ratios (aORs) of reporting use of patient portals from a multivariable logistic regression model, conditional on mothers’ motivation and concerns and all other characteristics.

^b^*P* values from Type 3 analysis based on the Wald test.

^c^Reference group does not have CI.

^d^HCP: health care practitioner.

### Mothers’ Willingness to Share Their Data From Different Sources

#### Association of Mothers’ Willingness to Share Data Through Electronic Medical Record Data and Independent Variables

The results of the bivariate analysis indicated that mothers’ willingness to share their electronic medical record (EMR) data was significantly related to mothers’ marital status (*P*=.01), household income (*P*=.04), child health status (*P*=.02), use of the internet to access health information (*P*=.02), motivation (*P*=.02), and privacy concerns (*P*=.02) about sharing health data (Multimedia Appendix 1).

When running the multivariate logistic regression analysis (Table 4), the variables that were statistically significantly associated with the willingness to share EMR data anonymously included mothers’ motivation to share data, mothers’ concern about sharing their health data, child health status, and HCP status. The results showed that mothers with a child in excellent-to-good health status (aOR 0.38, 95% CI 0.15-0.93) were less likely to share EMR data anonymously. Furthermore, mothers with one HCP (aOR 1.61, 95% CI 0.95-2.73) increased their likelihood of willingness to share their EMR data with researchers by 2.7 times. Additionally, very motivated mothers were nearly four times more willing to share their EMR data with researchers (aOR 3.64, 95% CI 2.00-6.63). Highly concerned mothers were 40% less likely to share EMR data with researchers, compared with mothers who were less concerned about sharing data.

#### Association of Mothers’ Willingness to Share Data Provided in a Web-Based Survey With Independent Variables

The bivariate analysis revealed that the status of HCP (*P*<.001), use of mobile phones (*P*=.01), use of the internet to access health information (*P*<.001), and mothers’ motivation to share data (*P*<.001) were statistically associated with mothers’ willingness to share their data in Web-based surveys (Multimedia Appendix 1). However, after adjusting for mothers’ characteristics in multivariate analysis, only the status of HCP (*P*<.001) and mothers’ motivation to share data (*P*<.001) were statistically associated with their willingness to share data through a Web-based survey (Table 4). Mothers who had 1 (aOR 4.22, 95% CI 1.97-9.05) or more than one HCP (aOR 4.47, 95% CI 1.94-10.3) were four times as likely to share their health data in Web-based surveys with researchers.

#### Association of Mothers’ Willingness to Share Data Entered in Health Apps or Collected From Devices and Independent Variables

The findings of this study indicated that the use of mobile phones (*P*<.001), use of the internet for health information (*P*<.001), mothers’ motivation to share data (*P*<.001), and mothers’ concerns about sharing data (*P*<.001) were statistically associated with their willingness to share mobile app data in bivariate analyses (Multimedia Appendix 1). The results from multivariate logistic regression analyses showed that younger mothers (aOR 1.93, 95% CI 1.12-3.32) were nearly two times more willing to share their app or device data with researchers than older mothers (Table 4). Mothers who had 1 (aOR 3.64, 95% CI 1.73-7.65) and more than one HCP (aOR 3.03, 95% CI 1.38-6.67) were three times more likely to share the health data of their apps or devices, compared with the reference group. Mothers who used smartphones (aOR 5.47, 95% CI 1.76-17) were five times more willing to share their data than the reference group.

#### Association of Mothers’ Willingness to Share GPS Location Data and Independent Variables

The independent variables of children’s age (*P*=.01), use of mobile phones (*P*=.03), access to health information (*P*<.001), mothers’ motivation (*P*<.001), and concerns (*P*<.001) were statistically associated with mothers’ willingness to share their GPS location in bivariate analyses (Multimedia Appendix 1).

**Table 4:** Results of multivariate logistic regression regarding mothers’ willingness to share different types of data with researchers and a set of predictors, including mothers’ demographic characteristics.

Effect			Willingness to share anonymous data from															
			Electronic medical data			Web-based surveys				Health app and device					GPS			
			aOR^a^ (95% CI)	*P* value^b^		aOR (95% CI)			*P* value^b^		aOR^a^ (95% CI)		*P* value^b^			aOR^a^ (95% CI)		*P* value^b^
**Age (years)**				**.52**					**.80**				**.02**					**.84**
	18-49		0.84 (0.49-1.43)			1.09 (0.57-2.07)					1.93 (1.12-3.32)					1.05 (0.63-1.76)		
	>50		1.00 (Reference)^c^			1.00 (Reference)^c^					1.00 (Reference)^c^					1.00 (Reference)^c^		
**Education level**				**.53**					**.14**				**.56**					**.84**
	4 or more years of college		1.14 (0.76-1.7)			0.68 (0.41-1.14)					1.13 (0.74-1.73)					1.04 (0.70-1.54)		
	Less than 4 years of a college degree		1.00 (Reference)^c^			1.00 (Reference)^c^					1.00 (Reference)^c^					1.00 (Reference)^c^		
**Occupational status**				**.53**					**.18**				**.84**					**.24**
	Employed		1.15 (0.74-1.81)			0.66 (0.36-1.21)					0.95 (0.59-1.53)					1.3 (0.84-2.02)		
	Unemployed		1.00 (Reference)^c^			1.00 (Reference)^c^					1.00 (Reference)^c^					1.00 (Reference)^c^		
**Marital status**				**.14**					**.66**				**.79**					**.82**
	Married		0.69 (0.42-1.12)			0.86 (0.44-1.68)					0.93 (0.53-1.63)					0.95 (0.59-1.53)		
	Unmarried		1.00 (Reference)^c^			1.00 (Reference)^c^					1.00 (Reference)^c^					1.00 (Reference)^c^		
**Income level (US $)**				**.16**					**.82**				**.32**					**.06**
		≥75,000	0.73 (0.48-1.13)			1.07 (0.61-1.86)					0.79 (0.49-1.27)					0.67 (0.44-1.01)		
		≤74,999	1.00 (Reference)^c^			1.00 (Reference)^c^					1.00 (Reference)^c^					1.00 (Reference)^c^		
**Health status**				**.23**					**.27**				**.59**					**.66**
	Excellent to good		0.67 (0.36-1.28)			1.57 (0.71-3.49)					1.21 (0.61-2.42)					0.87 (0.46-1.62)		
	Fair to poor		1.00 (Reference)^c^			1.00 (Reference)^c^					1.00 (Reference)^c^					1.00 (Reference)^c^		
**Child health status**				**.03**					**.51**									**.28**
	Excellent to good		0.38 (0.15-0.93)			1.51 (0.45-5.12)					1.31 (0.5-3.48)		.58			0.61 (0.25-1.5)		
	Fair to poor		1.00 (Reference)^c^			1.00 (Reference)^c^					1.00 (Reference)^c^		.59			1.00 (Reference)^c^		
**HCP^d^ status**				**.06**					**<.001**				**.003**					**.18**
	More than one HCP		2.23 (0.92-5.43)			4.47 (1.94-10.3)					3.03 (1.38-6.67)					2.11 (0.93-4.8)		
	Just one HCP		2.69 (1.15-6.27)			4.22 (1.97-9.05)					3.64 (1.73-7.65)					2.04 (0.93-4.5)		
	No HCP		1.00 (Reference)^c^			1.00 (Reference)^c^					1.00 (Reference)^c^					1.00 (Reference)^c^		
**Children’s age (years)**				**.07**					**.37**				**.94**					**.07**
	≤14		1.61 (0.95-2.73)			1.35 (0.7-2.6)					1.02 (0.58-1.79)					1.6 (0.96-2.66)		
	≥15		1.00 (Reference)^c^			1.00 (Reference)^c^					1.00 (Reference)^c^					1.00 (Reference)^c^		
**Number of children**				**.41**					**.78**				**.84**					**.06**
	More than one child		0.84 (0.55-1.28)			0.92 (0.53-1.61)					0.95 (0.6-1.52)					0.68 (0.44-1.02)		
	One child		1.00 (Reference)^c^			1.00 (Reference)^c^					1.00 (Reference)^c^					1.00 (Reference)^c^		
**Use of mobile phones**				**.90**					**.06**				**<.001**					**.22**
	Yes		1.07 (0.36-3.19)			2.85 (0.97-8.41)					5.47 (1.76-17)					2.01 (0.63-6.94)		
	No		1.00 (Reference)^c^			1.00 (Reference)^c^					1.00 (Reference)^c^					1.00 (Reference)^c^		
**Use of the internet to access health information**				**.61**					**.70**				**.69**					**.44**
	Yes		1.58 (0.27-9.27)			1.37 (0.28-6.7)					0.73 (0.16-3.4)					1.93 (0.35-10.69)		
	No		1.00 (Reference)^c^			1.00 (Reference)^c^					1.00 (Reference)^c^					1.00 (Reference)^c^		
**Mothers’ motivation to share their data**				**<.001**					**<.001**				**<.001**					**<.001**
	Somewhat motivated		2.42 (1.6-3.67)			5.94 (3.15-11.2)					3.37 (2.16-5.26)					3.1 (2.08-4.60)		
	Very motivated		3.64 (2.00-6.63)			2.87 (1.18-6.94)					5.30 (2.32-12.1)					5.15 (2.83-9.38)		
	Less motivated		1.00 (Reference)^c^			1.00 (Reference)^c^					1.00 (Reference)^c^					1.00 (Reference)^c^		
**Mothers’ concern about sharing health data**				**.008**					**.31**				**.09**					**<.001**
	Somewhat concerned		0.59 (0.35-1.00)			0.75 (0.34-1.68)					0.88 (0.47-1.68)					0.50 (0.27 0.77)		
	Very concerned		0.43 (0.25-0.73)			0.57 (0.26-1.25)					0.58 (0.31-1.08)					0.40 (0.22-0.60)		
	Less concerned		1.00 (Reference)^c^			1.00 (Reference)^c^					1.00 (Reference)^c^					1.00 (Reference)^c^		

^a^Adjusted odds ratios (aORs) of reporting willingness to share different type of data from a multivariable logistic regression model, conditional on mothers’ motivation and concerns and all other characteristics.

^b^*P* values from Type 3 analysis based on the Wald test.

^c^Reference group does not have CI.

^d^HCP: health care provider.

When adjusting for mothers’ characteristics through multivariate logistic regression, only mothers’ motivation to share data (*P*<.001) and mothers’ concerns with sharing data (*P*<.001) were statistically associated with their willingness to share their GPS location (Table 4). Furthermore, very motivated mothers (aOR 5.1, 95% CI 2.83-9.38) were five times more likely to be willing to share their GPS location data. Similarly, somewhat motivated mothers (aOR 3.1, 95% CI 2.08-4.60) were three times more willing to share their GPS location data than the reference group. Conversely, very concerned mothers (aOR 0.4, 95% CI 0.22-0.60) were 40% less willing to share their data, compared with less concerned ones.

## Discussion

This study explored mothers’ motivation to share health data, concerns with potential misuse of personal health information, and willingness to share different types of data with researchers, their utilization of patient portals, and their desire to keep their health information anonymous.

### Motivation to Share Data and Concern With Data Use

Our study results revealed that about half of the mothers were less motivated to share their data with researchers. Our results contradict the findings of a previous study that found that more than 78% of the surveyed respondents were more willing to share their data with researchers [25]. However, when we investigated the reasons driving these mothers to share their health data, we found that they were motivated to contribute to science and benefit other patients. On the other hand, we found that 55% of mothers were concerned with the misuse of their personal health information. A major concern with the misuse of personal health data has been reported among 68% of healthy volunteers in survey studies [28]. Further analysis showed that their privacy concerns were related to data misuse, especially the risk that their data would be stolen or used without their consent. In other words, their concern impacted their perceived risk of privacy for disclosing their data, which is consistent with previous studies [18].

### Desire to Keep Health Information Anonymous and Use of Patient Portal

Our findings on the respondents’ desire to keep their health information anonymous have also been reported elsewhere [29]. Moreover, we found that mothers’ desire to keep their personal health information anonymous was dependent on their perceived concerns. These results were in line with previous studies on the benefits and concerns of data sharing [25].

We found that a relatively high proportion of patient portal use (63%) among women in the general population may seem unusual. Although health portal use by patients is becoming more prevalent, a recent study estimated that only 32% of outpatients of a Dutch academic health center used a patient portal [30]. Another study reported a 58% registration rate to the patient portal among older adult patients linked to an academic medical center in the United States [31]. However, more recent studies have shown a much higher percentage of portal users among adult patients (82%) in the United States [32]. In addition, another study identified that 34% of Americans have been offered access to patient health information through an HCP, but only 28% accessed this information [33].

### Willingness to Share Anonymous Data

Our study found that the majority of the mothers were not willing to share anonymous data from medical records and their GPS location using their mobile app (or device). However, these mothers were willing to share anonymous data through a Web-based survey. Our review of the literature cannot corroborate these findings as most of the studies on individuals’ willingness to share their health data focused on health information exchanges [11,12,14-16,18-21]. These studies found that data security and privacy preferences shape consumers’ attitudes toward health information exchanges. Moreover, consumers perceived health information exchanges to confer benefits, such as better coordination of care [15] and improved health care quality [11]. It has been reported that a person’s willingness to share health data is directly associated with the subject suffering from progressive or chronic illness [34]. However, our respondents’ or their children’s health status were not associated with their willingness to share data. Other studies on sharing patient health data reported conflicting results. One strand of studies has shown that individuals are willing to share their data to benefit health outcomes [35], whereas another strand found that anonymity, research use, engagement with a trusted entity, transparency to access medical records, and incentives affected individuals’ willingness to share their data [22,29]. The findings of this study confirmed the latter strand, indicating that the level of mothers’ concerns played a more significant role than their motivations in sharing their medical records. In fact, mothers were concerned that their data would be misused or stolen. With regard to participants sharing GPS location, studies have reported low willingness to share location data [36,37]. The results from these two studies conform with our findings, and according to a study report, sharing the GPS location can jeopardize the privacy and personal information of patients [38].

### Strengths and Limitations

To our knowledge, this is the first study that employed the opt-in panel to assess non-Hispanic white mothers’ attitudes and perceptions toward data sharing [4]. As the opt-in panel is a self-selected sample of women, matched to the background of mothers of children with autism, our results about health data sharing behavior can only be generalized to non-Hispanic white mothers in the same age, education, and employment status group of our study sample. Owing to this study design, our findings are not representative of nonwhite single women in the United States or other populations in the United States. Moreover, we should interpret our results with caution when compared with similar studies due to the nature of our survey questions. In particular, previous studies have framed patient health data entirely from the health information exchange perspective, and more specifically, those studies investigated data sharing with care providers but not with researchers. Finally, the obtained results of some of our statistical analyses were too small; therefore, statistical testing can be unstable.

### Future Research Implications

In an era dominated by mobile apps and wearable devices, researchers should focus on the value of the privacy calculus in the context of data sharing for research [26,39,40]. First, this interest is rising with the spread of ubiquitous computing and unlimited options for collecting, processing, distributing, and using data, which can overwhelm participants’ interest in sharing their data. On the other hand, the future of health care discoveries rests upon the amount of data collected from patients. In this regard, many participants may not know how their data have been used and accessed. Therefore, this lack of clear communication among members of the research community and the general population on how data are being collected and used may raise ethical issues related to data sharing [23].

Second, to facilitate and improve participation in citizen research, which requires recruiting a large number of individuals to participate in a health research study, a priori market segmentation studies should be implemented to assess consumers’ data-sharing behavior. Such methods are more rigorous than extrapolating the findings from the general population. Consumers’ data-sharing behavior is warranted in part because of the digital divide that is due to the difference in socioeconomic status exhibited within the general population [11,33,41,42]. Previous studies have suggested that increasing consumers’ trust in information communication technology and data sharing can lead to higher participation in data-sharing research [18]. The findings of this study suggest that researchers studying data-sharing behavior should have a better understanding of their targeted group so that they can identify strategies to increase their participation. Furthermore, our findings suggest the need to engage patients in addressing the underlying reasons for their concerns. Finally, our findings are aligned with previous research that recommended assessing consumers’ data-sharing behavior. This assessment can provide guidelines for Web and apps development that can provide consumers with better access and control over their data, which can subsequently increase consumers’ trust [18,22].

## Appendix

Multimedia Appendix 1 Supplemental results (bivariate analysis).

## Abbreviations

aOR: adjusted odds ratio
ASD: autism spectrum disorder
eHealth: electronic health
EMR: electronic medical record
HCP: health care provider
IAN: Interactive Autism Network
OR: odds ratio
