# Correction: Non-Hispanic White Mothers’ Willingness to Share Personal Health Data With Researchers: Survey Results From an Opt-in Panel

**DOI:** 10.2196/24183

**Published:** 2020-11-03

**Authors:** Adam Bouras, Eduardo J Simoes, Suzanne Boren, Lanis Hicks, Iris Zachary, Christoph Buck, Satvinder Dhingra, Richard Ellis

**Affiliations:** 1 Department of Health Management and Informatics University of Missouri-Columbia Columbia, MO United States; 2 Missouri University Institute for Data Science and Informatics University of Missouri-Columbia Columbia, MO United States; 3 Centre for Future Enterprise QUT Business School Queensland University of Technology Brisbane Australia; 4 Public Good Venture Limited Atlanta, GA United States

In “Non-Hispanic White Mothers’ Willingness to Share Personal Health Data With Researchers: Survey Results From an Opt-in Panel” (J Participat Med 2020;12(2):e14062) the authors noted three errors.

One co-author, Cristoph Buck, was not included in the author list of the originally published manuscript. As well, the name of author Eduardo J Simoes was originally listed as "Eduardo Simoes". Authors were listed as follows on the published manuscript:

Adam Bouras^1,2^, MSHI, MHA, MSc; Eduardo Simoes^1,2^, MD, MPH; Suzanne Boren^1,2^, PhD; Lanis Hicks^1,2^, PhD; Iris Zachary^1^, PhD; Satvinder Dhingra^3^, MPH; Richard Ellis^3^, BSc

This was incomplete, and has been corrected to:

Adam Bouras^1,2^, MSHI, MHA, MSc; Eduardo J Simoes^1,2^, MD, MPH; Suzanne Boren^1,2^, PhD; Lanis Hicks^1,2^, PhD; Iris Zachary^1^, PhD; Christoph Buck^3^, PhD; Satvinder Dhingra^4^, MPH; Richard Ellis^4^, BSc

Cristoph Buck's affiliation is as follows:

Centre for Future Enterprise, QUT Business School, Queensland University of Technology, Brisbane, Australia

This affiliation is listed as affiliation 3 in the corrected manuscript, and the previously listed affiliation 3 (for authors Satvinder Dhingra and Richard Ellis) is renumbered to affiliation 4. Affiliations 1 and 2 remain unchanged.

Additionally, the original manuscript was missing the following funding statement:

The work of EJS was made possible with support from Washington University in St. Louis CDTR (Grant Number P30DK092950 from the NIDDK). The content is solely the responsibility of the authors and does not necessarily represent the official views of the CDTR or NIDDK.

An "Acknowledgments" section containing this statement is included in the corrected manuscript.

The correction will appear in the online version of the paper on the JMIR Publications website on November 3, 2020, together with the publication of this correction notice. Because this was made after submission to PubMed, PubMed Central, and other full-text repositories, the corrected article has also been resubmitted to those repositories.

