# Supplementary material for: Non-Hispanic White Mothers’ Willingness to Share Personal Health Data With Researchers: Survey Results From an Opt-in Panel
Source: J Particip Med. 2020 May 15;12(2):e14062. doi: 10.2196/14062 (PMC7434052; doi:10.2196/14062)
Supplement: Multimedia Appendix 1 [file jopm_v12i2e14062_app1.docx]

| Table 1: Frequency of respondents’ desire to keep their health information anonymous according to mothers’ demographics, children and health domain characteristics. | | | | | |
| --- | --- | --- | --- | --- | --- |
| Independent variables | | Not at all important | Somewhat important | Extremely important | P-value |
|  |  | N (%) | N (%) | N (%) |  |
| Age group | 18-49 | 11 (1.77%) | 123 (19.77%) | 310 (49.84%) | 0.48 |
|  | 50+ | 2 (0.32%) | 46 (7.4%) | 130 (20.9%) |  |
| Education | 2-year college degree or less | 9 (1.45%) | 68 (10.93%) | 198 (31.83%) | 0.11 |
|  | 4-year college degree and more | 4 (0.64%) | 101 (16.24%) | 242 (38.91%) |  |
| Employment status | Employed or self-employed | 10 (1.61%) | 119 (19.13%) | 323 (51.93%) | 0.71 |
|  | Other employment status | 3 (0.48%) | 50 (8.04%) | 117 (18.81%) |  |
| Marital status | Married | 9 (1.45%) | 131 (21.06%) | 345 (55.47%) | 0.72 |
|  | Other marital status | 4 (0.64%) | 38 (6.11%) | 95 (15.27%) |  |
| Household income | ≤$74,999 | 6 (0.96%) | 66 (10.61%) | 162 (26.05%) | 0.71 |
|  | ≥$75,000 | 7 (1.13%) | 103 (16.56%) | 278 (44.69%) |  |
| Mother health status | Fair-to-Poor | 1 (0.16%) | 15 (2.41%) | 46 (7.4%) | 0.81 |
|  | Excellent-to-Good | 12 (1.93%) | 154 (24.76%) | 394 (63.34%) |  |
| Child health status | Fair-to-Poor | 0 (0%) | 6 (0.96%) | 22 (3.54%) | 0.54 |
|  | Excellent-to-Good | 13 (2.09%) | 163 (26.21%) | 418 (67.2%) |  |
| Health care provider status | I don’t have an HCP | 4 (0.64%) | 9 (1.45%) | 39 (6.27%) | 0.02 |
|  | Have more than one HCP | 4 (0.64%) | 50 (8.04%) | 136 (21.86%) |  |
|  | Yes, just one HCP | 5 (0.8%) | 110 (17.68%) | 265 (42.6%) |  |
| Children age | 14 and younger | 9 (1.45%) | 107 (17.2%) | 265 (42.6%) | 0.66 |
|  | 15 and older | 4 (0.64%) | 62 (9.97%) | 175 (28.14%) |  |
| Number of children | One child | 6 (0.96%) | 88 (14.15%) | 232 (37.3%) | 0.89 |
|  | More than one child | 7 (1.13%) | 81 (13.02%) | 208 (33.44%) |  |
| Use of mobile phone | Yes | 10 (1.74%) | 148 (25.78%) | 395 (68.82%) | 0.15 |
|  | No | 2 (0.35%) | 7 (1.22%) | 12 (2.09%) |  |
| Use of the internet to access health information | Yes | 5 (0.82%) | 112 (18.3%) | 244 (39.87%) | 0.03 |
|  | No | 8 (1.31%) | 56 (9.15%) | 187 (30.56%) |  |
| Mothers motivations for willing to share their data | Less motivated | 7 (1.13%) | 63 (10.13%) | 232 (37.3%) | 0.01 |
|  | Somewhat motivated | 4 (0.64%) | 79 (12.7%) | 165 (26.53%) |  |
|  | Very motivated | 2 (0.32%) | 27 (4.34%) | 43 (6.91%) |  |
| Mothers Concerns that personal health information will be misused | Less concerned | 10 (1.61%) | 52 (8.36%) | 53 (8.52%) | <.001 |
|  | Somewhat concerned | 2 (0.32%) | 67 (10.77%) | 158 (25.4%) |  |
|  | Very concerned | 1 (0.16%) | 50 (8.04%) | 229 (36.82%) |  |
| % cell | | | | | |

| Table 2: Frequency of respondents’ use of patient portal according to mothers’ demographics, children, and health domain characteristics | | | | |
| --- | --- | --- | --- | --- |
|  | | Use of Patient Portal | | |
|  |  | Never heard of it or used it | Used it more than once a month | P-value |
|  |  | N (%) | N (%) |  |
| Age group | 18-49 | 163 (26.21%) | 281 (45.18%) | 0.96 |
|  | 50+ | 65 (10.45%) | 113 (18.17%) |  |
| Education | 2-year college degree or less | 103 (16.56%) | 172 (27.65%) | 0.71 |
|  | 4-year college degree and more | 125 (20.1%) | 222 (35.69%) |  |
| Employment status | Employed or self-employed | 167 (26.85%) | 285 (45.82%) | 0.81 |
|  | Other employment status | 61 (9.81%) | 109 (17.52%) |  |
| Marital status | Married | 176 (28.3%) | 309 (49.68%) | 0.72 |
|  | Other marital status | 52 (8.36%) | 85 (13.67%) |  |
| Household income | ≤$74,999 | 96 (15.43%) | 138 (22.19%) | 0.08 |
|  | ≥$75,000 | 132 (21.22%) | 256 (41.16%) |  |
| Mother health status | Fair-to-Poor | 23 (3.7%) | 39 (6.27%) | 0.94 |
|  | Excellent-to-Good | 205 (32.96%) | 355 (57.07%) |  |
| Child health status | Fair-to-Poor | 5 (0.8%) | 23 (3.7%) | 0.03 |
|  | Excellent-to-Good | 223 (35.85%) | 371 (59.65%) |  |
| Health care provider status | I don’t have an HCP | 35 (5.63%) | 17 (2.73%) | <.001 |
|  | Have more than one HCP | 59 (9.49%) | 131 (21.06%) |  |
|  | Yes, just one HCP | 134 (21.54%) | 246 (39.55%) |  |
| Children age | 14 and younger | 136 (21.86%) | 245 (39.39%) | 0.53 |
|  | 15 and older | 92 (14.79%) | 149 (23.95%) |  |
| Number of children | One child | 128 (20.58%) | 198 (31.83%) | 0.16 |
|  | More than one child | 100 (16.08%) | 196 (31.51%) |  |
| Use of mobile phone | Yes | 189 (32.93%) | 364 (63.41%) | <.001 |
|  | No | 12 (2.09%) | 9 (1.57%) |  |
| Use of the internet to access health information | Yes | 82 (13.4%) | 279 (45.59%) | <.001 |
|  | No | 140 (22.88%) | 111 (18.14%) |  |
| Mothers motivations for willing to share their data | Less motivated | 137 (22.03%) | 165 (26.53%) | <.001 |
|  | Somewhat motivated | 71 (11.41%) | 177 (28.46%) |  |
|  | Very motivated | 20 (3.22%) | 52 (8.36%) |  |
| Mothers Concerns that their personal health information will be misused | Less concerned | 46 (7.4%) | 69 (11.09%) | 0.58 |
|  | Somewhat concerned | 78 (12.54%) | 149 (23.95%) |  |
|  | Very concerned | 104 (16.72%) | 176 (28.3%) |  |
| % cell | | | | |

| Table 3: Frequency of respondents’ willingness to share four types of health information data with researchers by mothers’ demographics, children, and health domain characteristics | | | | | | | | | | | | | |
| --- | --- | --- | --- | --- | --- | --- | --- | --- | --- | --- | --- | --- | --- |
|  | | Data from medical  records | | | Data provided directly by completing an online survey | | | Data entered or collected through the mobile app | | | GPS location data from a mobile device | | |
|  |  | Yes | No | P-value | Yes | No | P-value | Yes | No | P-value | Yes | No | P-value |
|  |  | N (%) | N (%) |  | N (%) | N (%) |  | N (%) | N (%) |  | N (%) | N (%) |  |
| Age group | 18-49 | 161 (25.88%) | 283 (45.5%) | 0.84 | 369 (59.32%) | 75 (12.06%) | 0.19 | 323 (51.93%) | 121 (19.45%) | <.001 | 190 (30.55%) | 254 (40.84%) | 0.07 |
|  | 50+ | 63 (10.13%) | 115 (18.49%) |  | 140 (22.51%) | 38  (6.11%) |  | 100 (16.08%) | 78 (12.54%) |  | 62 (9.97%) | 116 (18.65%) |  |
| Education | 2-year college degree or less | 97 (15.59%) | 178 (28.62%) | 0.73 | 229 (36.82%) | 46 (7.4%) | 0.41 | 181 (29.1%) | 94 (15.11%) | 0.30 | 106 (17.04%) | 169 (27.17%) | 0.37 |
|  | 4-year college degree and more | 127 (20.42%) | 220 (35.37%) |  | 280 (45.02%) | 67 (10.77%) |  | 242 (38.91%) | 105 (16.88%) |  | 146 (23.47%) | 201 (32.32%) |  |
| Employment status | Employed or self-employed | 165 (26.53%) | 287 (46.14%) | 0.68 | 365 (58.68%) | 87 (13.99%) | 0.25 | 307 (49.36%) | 145 (23.31%) | 0.94 | 189 (30.39%) | 263 (42.28%) | 0.28 |
|  | Other employment status | 59 (9.49%) | 111 (17.85%) |  | 144 (23.15%) | 26 (4.18%) |  | 116 (18.65%) | 54 (8.68%) |  | 63 (10.13%) | 107 (17.2%) |  |
| Marital status | Married | 162 (26.05%) | 323 (51.93%) | 0.01 | 395 (63.5%) | 90 (14.47%) | 0.64 | 325 (52.25%) | 160 (25.72%) | 0.32 | 191 (30.71%) | 294 (47.27%) | 0.28 |
|  | Other marital status | 62 (9.97%) | 75 (12.06%) |  | 114 (18.33%) | 23 (3.7%) |  | 98 (15.76%) | 39 (6.27%) |  | 61 (9.81%) | 76 (12.22%) |  |
| Household income | ≤$74,999 | 96 (15.43%) | 138 (22.19%) | 0.04 | 192 (30.87%) | 42 (6.75%) | 0.91 | 163 (26.21%) | 71 (11.41%) | 0.49 | 105 (16.88%) | 129 (20.74%) | 0.09 |
|  | ≥$75,000 | 128 (20.58%) | 260 (41.8%) |  | 317 (50.96%) | 71 (11.41%) |  | 260 (41.8%) | 128 (20.58%) |  | 147 (23.63%) | 241 (38.75%) |  |
| Mother health status | Fair-to-Poor | 27 (4.34%) | 35 (5.63%) | 0.19 | 48 (7.72%) | 14 (2.25%) | 0.34 | 41 (6.59%) | 21 (3.38%) | 0.74 | 27 (4.34%) | 35 (5.63%) | 0.61 |
|  | Excellent-to-Good | 197 (31.67%) | 363 (58.36%) |  | 461 (74.12%) | 99 (15.92%) |  | 382 (61.41%) | 178 (28.62%) |  | 225 (36.17%) | 335 (53.86%) |  |
| Child health status | Fair-to-Poor | 16 (2.57%) | 12 (1.93%) | 0.02 | 23 (3.7%) | 5 (0.8%) | 0.97 | 18 (2.89%) | 10 (1.61%) | 0.67 | 15 (2.41%) | 13 (2.09%) | 0.15 |
|  | Excellent-to-Good | 208 (33.44%) | 386 (62.06%) |  | 486 (78.14%) | 108 (17.36%) |  | 405 (65.11%) | 189 (30.39%) |  | 237 (38.1%) | 357 (57.4%) |  |
| Health care provider status | I don’t have an HCP | 11 (1.77%) | 41 (6.59%) | 0.06 | 29 (4.66%) | 23 (3.7%) | <.001 | 23 (3.7%) | 29 (4.66%) | <.001 | 13 (2.09%) | 39 (6.27%) | 0.06 |
|  | Have more than one HCP | 69 (11.09%) | 121 (19.45%) |  | 161 (25.88%) | 29 (4.66%) |  | 131 (21.06%) | 59 (9.49%) |  | 80 (12.86%) | 110 (17.68%) |  |
|  | Yes, just one HCP | 144 (23.15%) | 236 (37.94%) |  | 319 (51.29%) | 61 (9.81%) |  | 269 (43.25%) | 111 (17.85%) |  | 159 (25.56%) | 221 (35.53%) |  |
| Children age | 14 and younger | 147 (23.63%) | 234 (37.62%) | 0.09 | 320 (51.45%) | 61 (9.81%) | 0.08 | 277 (44.53%) | 104 (16.72%) | <.001 | 82 (13.18%) | 159 (25.56%) | 0.01 |
|  | 15 and older | 77 (12.38%) | 164 (26.37%) |  | 189 (30.39%) | 52 (8.36%) |  | 146 (23.47%) | 95 (15.27%) |  | 170 (27.33%) | 211 (33.92%) |  |
| Number of children | One child | 120 (19.29%) | 206 (33.12%) | 0.66 | 243 (39.07%) | 53 (8.52%) | 0.87 | 216 (34.73%) | 110 (17.68%) | 0.33 | 115 (18.49%) | 181 (29.1%) | 0.42 |
|  | More than one child | 104 (16.72%) | 192 (30.87%) |  | 266 (42.77%) | 60 (9.65%) |  | 207 (33.28%) | 89 (14.31%) |  | 137 (22.03%) | 189 (30.39%) |  |
| Use of mobile phone | Yes | 201 (35.02%) | 352 (61.32%) | 0.76 | 460 (80.14%) | 93 (16.2%) | 0.01 | 393 (68.47%) | 160 (27.87%) | <.001 | 234 (40.77%) | 319 (55.57%) | 0.03 |
|  | No | 6 (1.05%) | 15 (2.61%) |  | 12 (2.09%) | 9 (1.57%) |  | 6 (1.05%) | 15 (2.61%) |  | 4 (0.7%) | 17 (2.96%) |  |
| Use of the internet to access health information | Yes | 154 (25.16%) | 207 (33.82%) | <.001 | 322 (52.61%) | 39 (6.37%) | <.001 | 287 (46.9%) | 74 (12.09%) | <.001 | 173 (28.27%) | 188 (30.72%) | <.001 |
|  | No | 68 (11.11%) | 183 (29.9%) |  | 180 (29.41%) | 71 (11.6%) |  | 130 (21.24%) | 121 (19.77%) |  | 77 (12.58%) | 174 (28.43%) |  |
| Mothers motivation for willing to share their data | Less motivated | 69 (11.09%) | 233 (37.46%) | <.001 | 212 (34.08%) | 90 (14.47%) | <.001 | 162 (26.05%) | 140 (22.51%) | <.001 | 78 (12.54%) | 224 (36.01%) | <.001 |
|  | Somewhat motivated | 113 (18.17%) | 135 (21.7%) |  | 232 (37.3%) | 16 (2.57%) |  | 198 (31.83%) | 50 (8.04%) |  | 128 (20.58%) | 120 (19.29%) |  |
|  | Very motivated | 42 (6.75%) | 30 (4.82%) |  | 65 (10.45%) | 7 (1.13%) |  | 63 (10.13%) | 9 (1.45%) |  | 46 (7.4%) | 26 (4.18%) |  |
| Mothers Concerns that personal health information will be misused | Less concerned | 60 (9.65%) | 55 (8.84%) | <.001 | 100 (16.08%) | 15 (2.41%) | 0.11 | 172 (27.65%) | 108 (17.36%) | <.001 | 67 (10.77%) | 48 (7.72%) | <.001 |
|  | Somewhat concerned | 82 (13.18%) | 145 (23.31%) |  | 189 (30.39%) | 38 (6.11%) |  | 163 (26.21%) | 64 (10.29%) |  | 91 (14.63%) | 136 (21.86%) |  |
|  | Very concerned | 82 (13.18%) | 198 (31.83%) |  | 220 (35.37%) | 60 (9.65%) |  | 88 (14.15%) | 27 (4.34%) |  | 94 (15.11%) | 186 (29.9%) |  |
| % cell | | | | | | | | | | | | | |
